# A tuberculosis contact investigation involving a large number of contacts tested with interferon-gamma release assay at a nursing school: Kanagawa, Japan, 2012

**DOI:** 10.5365/wpsar.2018.9.1.001

**Published:** 2018-08-06

**Authors:** Masako Tasaka, Tamae Shimamura, Mami Iwata, Takahiro Toyozawa, Masaki Ota

**Affiliations:** a Kanagawa Prefectural Institute of Public Health, Kanagawa, Japan.; b Research Institute of Tuberculosis, Tokyo, Japan.; c Naka Welfare and Health Office, Yokohama, Japan.; d Yokohama City Health Department, Yokohama, Japan.

## Abstract

In May 2012, a teacher of a nursing school with about 300 staff members and students in Japan was diagnosed with sputum smear-positive pulmonary tuberculosis (TB), leading to an investigation involving nearly 300 contacts. We describe the contacts’ closeness to the index TB patient and the likelihood of TB infection and disease.

A case of TB was defined as an individual with positive bacteriological tests or by a physician diagnosis of TB. A latent TB infection (LTBI) case was defined as an individual who had a positive interferon-gamma release assay (IGRA).

A total of 283 persons screened with IGRA were analysed. Eight persons (2.8%, 95% confidence interval [CI]: 1.2–5.4) tested positive by IGRA; one student who had intermediate (less than 10 hours) contact with the index patient was found to have pulmonary TB by chest X-ray. The positivity in IGRA among staff members with very close contact with the index patient (4 of 21, 19%, 95% CI: 5.4–42%) with a statistically significant relative risk of 17 (95% CI: 2.0–140) was high compared with that of the intermediate contacts (1 of 88, 1.1% [95% CI: 0.028–6.2]). There was a statistically significant trend in the risk of TB infection and closeness with the index patient among the staff members and students (*P* < 0.00022).

In congregate settings such as schools, the scope of contact investigation may have to be expanded to detect a TB case among those who had brief contact with the index patient.

## Introduction

In Japan, the tuberculosis (TB) notification rate has declined in the past six decades from 698.4 per 100 000 population in 1951 to 17.7 per 100 000 population in 2011. (*1*) However, 8000 smear-positive TB cases are still reported annually, and more than 65% of those involve persons aged 65 years or older. (*2*) TB outbreaks involving hospitals, workplaces and homeless people have also been reported; (*3*–*5*) however, only a few involving schools were reported in the past decade. (*6*, *7*)

In May 2012, a teacher in her 50s was diagnosed with sputum smear-positive pulmonary tuberculosis (TB). She taught at a nursing school in Kanagawa, Japan that has over 300 staff members and students. At the school, new teachers and students are tested with tuberculin skin testing (TST), followed by interferon-gamma release assay (IGRA), if indicated, and annual chest X-ray (CXR) thereafter. Although the teacher had a cough for several months and had an abnormal finding by CXR a year before, TB had not been suspected since she had a history of asthma and nontuberculous mycobacterial infection. The teacher had close contact with other teachers and students, particularly those in the first and second years. The affiliated hospital demanded all the staff and students be screened for TB, which led to an unusually large-scale contact investigation involving over 300 individuals.

TB contact investigations in Japan rely on IGRA (*1*, *6*, *8*) rather than TST to screen latent TB infection (LTBI). IGRA is more specific and avoids interference caused by Bacillus Calmette–Guérin (BCG) vaccination, (*9*) which an estimated 90–95% of the population receives. (*10*) This study aims to describe and compare the contacts’ closeness to the index TB patient with the positivity of IGRA among those contacts.

## Methods

A case of TB was defined as an individual who was confirmed with a positive sputum smear, culture or nucleic acid amplification test (NAAT) or by a physician diagnosis of TB from January 2011 through December 2013. An LTBI case was defined as an individual who had a positive IGRA.

The index patient’s period of infectiousness was determined to be from December 2011 through May 2012 based on her history of symptoms.

We conducted a retrospective cohort study, enrolling almost all staff members and nursing students who were considered to have had contact with the index TB patient. The following three groups were excluded from the analysis: 1) staff and students who had a history of TB, 2) students who had a history of IGRA positivity or LTBI treatment at or before entry to the nursing school, and 3) staff who had been working more than three years at the school and had a history of IGRA positivity or LTBI treatment before 2009. For these groups, it was impossible to attribute their IGRA positivity to contact with the index patient in this investigation, and it was unlikely that the previous events were linked to this investigation. Since our focus of this study was to compare the contacts’ closeness to the index TB patient with their IGRA results, those who were screened solely by CXR (mostly administrative staff who did not have many contacts with the index patient) were also excluded from the analysis.

The contacts were divided into four groups: very close contacts (the staff who shared the same room with the index patient), close contacts (first- and second-year students who attended the class of the index patient for over 10 hours during the infectious period), intermediate contacts (third-year students who attended the index patient’s class for 10 hours or less) and other contacts (remaining staff and students who had almost no contact with the index patient). The very close contacts were tested twice with IGRA in May and July–August 2012; the close, the intermediate and the other contacts were tested once with IGRA in July–August 2012. However, 19 of the other contacts, mostly administrative staff, who did not have contact with the index patient were tested only by CXR in May 2012. All the IGRA-positive contacts were screened by CXR, and people who had abnormal findings were referred to a chest physician for follow-up. Those who were IGRA-positive without any abnormal findings on CXR were treated for LTBI with isoniazid for six to nine months. The one who was diagnosed with pulmonary TB was tested (sputum acid-fast bacilli smear and culture three times and NAAT) and treated with the standard regimen.

Analysis of IGRA positivity, 95% confidence intervals, and other statistical tests were carried out with R software (The R Foundation, Vienna, Austria). A Fisher’s exact test was used to calculate the relative risks among the contact groups. The intermediate contact group was used as the reference in calculating the relative risks because they were a large group (about 90 individuals) and were less likely to be exposed to the index patient but unlikely zero positivity. A Cochran-Armitage test was conducted to determine whether there was a trend among the positivity of the groups. A *p*-value less than 0.05 was considered statistically significant.

### Ethics

This investigation was conducted in accordance with the Infectious Disease Control Act of 1999 of Japan. We also obtained a waiver of ethical review for the study from the Institutional Review Board of the Research Institute of Tuberculosis because this study was retrospective, it relied on secondary use of the data that had been already collected by the local health offices, and it did not involve confidential information.

## Results

**Fig. 1:**
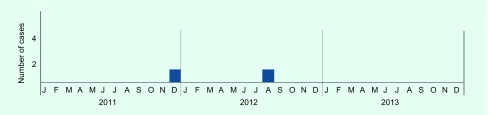
Epidemiologic curve of TB cases at a nursing school in Kanagawa, Japan, by month of symptom onset or diagnosis (if asymptomatic), 2011–2013

A total of 307 persons were enrolled as contacts for screening (**Table 1**) either with IGRA (285 contacts, 93%) or CXR (134 contacts, 44%) or both (115 contacts, 37%). Three first-year students had already tested positive by IGRA and had CXR at entry in early April 2012, and they were excluded from the initial screening. These three students were continuously followed up with CXR every six months. The 19 staff members (6%) who were screened only by CXR were excluded from the analysis. Of the 285 tested by IGRA, one teacher and one student were also excluded since they had histories of TB (about 14 years before) and LTBI treatment, respectively, before the event. Of the 283 (100%) who were analysed, eight (2.8%, 95% confidence interval [CI]: 1.2–5.4) were positive by IGRA (**Table 2**). Of those eight, four staff members and three second-year students tested positive by IGRA in May; one third-year student tested positive in July 2012. The age groups of the IGRA-positive students and the staff members were 20–29 years and 40–49 years, respectively. One third-year IGRA-positive student was found by CXR in August 2012 to have pulmonary TB; the student was smear- and culture-negative (epidemic curve in **Fig. 1**). The student had CXR in April 2012 for the routine check-up and also in May 2012 for contact investigation, and both were considered normal. No other staff member or student developed active pulmonary TB.

**Table 1:** Characteristics of the TB contacts of a nursing school and types of screening tests conducted, Kanagawa, Japan, 2011–2013

-	Staff	Students				Total
		first-year	second-year	third-year	Other	
**Number**	**56**	**81**	**76**	**88**	**6**	**307**
**Age (Median, IQR)**	**46 (10.5)**	**18 (1)**	**19 (1)**	**21 (2)**	**32 (15)**	**-**
**Female (%)**	**31 (55)**	**76 (94)**	**70 (92)**	**80 (91)**	**5 (83)**	**262 (85)**
**IGRA done**	**37**	78*	**76**	**88**	**6**	**285**
**Chest X-ray taken**	**44**	**0**	**0**	**88**	**2**	**134**

IGRA = interferon-gamma release assay

IQR = interquartile range

* Three students had already tested positive by IGRA at entry to the school and were excluded from the analysis.

**Table 2:** Numbers of persons with TB disease and with positive IGRA test among staff and students of a nursing school in relation to a TB contact investigation, Kanagawa, Japan, 2011–2013

-	TB disease		Persons with IGRA-positive test, including TB disease			Population
	n	% (95% CI)	n	% (95% CI)	RR^†^ (95% CI)	n
**Very close contacts**						
**Staff shared room with index patient**	**0**	**0 (0–16)**	**4**	**19 (5.4–42)**	**17 (2.0–140)**	**21**
**Close contacts**						
**second-year students**	**0**	**0 (0–4.7)**	**3^‡^**	**4.0 (0.83–11.2)**	**3.5 (0.37–33.1)**	**75^‡^**
**first-year students**	**0**	**0 (0–4.6)**	0**	**0 (0–4.6)**	**n/a**	78**
**Intermediate contacts**						
**third-year students**	**1**	**1.1 (0.028–6.2)**	**1**	**1.1 (0.0028–6.2)**	**1**	**88**
**Other contacts**						
**Staff in other rooms**	**0**	**0 (0–20)**	0*	**0 (0–20)**	**n/a**	15*
**Other students**	**0**	**0 (0–46)**	**0**	**0 (0–46)**	**n/a**	**6**
**Total**	**1**	**0.35 (0.0089–2.0)**	**8**	**2.8 (1.2–5.4)**	**-**	**283**

LTBI = latent tuberculosis infection, CI = confidence interval, n/a = not available, RR = relative risk, TB = tuberculosis

^†^ Compared with third-year students.

^‡^ Another student had a positive result; however, he was excluded from analysis because he had a history of LTBI treatment before entry to the school.

* Another staff member had a positive result; however, she was excluded from the analysis because she had a history of TB treatment 14 years before the current event.

** An additional three students had already tested positive for IGRA at entry to the school and were excluded from the analysis.

The Cochran-Armitage test revealed that there was a statistically significant trend in the risk of developing TB or LTBI among the ranked groups of staff members and students (***P*** = 0.00022).

Before 2011, no staff members or students developed tuberculosis except one teacher who developed pulmonary tuberculosis in 1998.

The highest prevalence of IGRA test positivity was found in the very close contacts who shared the same office (19% [95% CI: 5.4–42%]), with a statistically significant relative risk of 17.0 (95% CI: 2.0–140), compared with the intermediate contacts group (1.1% [95% CI: 0.028–6.2%]) (**Table 2**). The Cochran-Armitage test revealed that there was a statistically significant trend in the risk of developing TB or LTBI among the ranked groups of staff members and students (*P* = 0.00022).

## Discussion

We conducted a TB contact investigation at a nursing school in Japan after a teacher was diagnosed with pulmonary TB. During the investigation, almost the entire staff and student body were screened for TB infection by IGRA, which makes the results of the investigation more accurate than when TST is used in settings with high BCG coverage such as in Japan. (*6*–*8*, *11*, *12*) The staff who shared an office with the index patient were 17 times more likely to have LTBI than the intermediate contacts consisting of third-year students. Although the staff members were older than the students, IGRA positivity among middle-aged Japanese men and women is not normally as high as 19% (e.g. 3.5% [95% CI: 1.6–6.6%] in the 35–54 year age group). (*8*) Also, there was a statistically significant trend in the risk of TB infection among the staff members and students ordered by duration of close contact, implying a dose–response relationship seen in previous studies. (*13*) These findings suggest that this event was a TB outbreak that was propagated from the index patient to staff members and students.

Of note, one of the intermediate contacts was not only infected with TB but also developed TB disease, suggesting contact of even fewer than 10 hours may result in TB infection. Thus, the scope of TB contact investigations may have to be expanded, (*14*) particularly in school settings. (*13*)

One weakness of this investigation is the lack of molecular data to confirm that the infective strains were related. Although a third-year student developed TB disease, the sputum culture was negative. However, considering the fact that 13% of all TB cases in Japan are bacteriologically negative, (*15*) this is not uncommon. The possibility that the student acquired the infection outside of the school is small, considering the low incidence of pulmonary TB in young Japanese (about 3 per 100 000 population in those aged from 15 to 24 years (*15*)) and the timing of his development of the disease at about eight months after the index patient.

Another limitation is that we were unable to take the baseline IGRA from the contacts except a few who had a large tuberculin reaction at routine entry screening and were retested with IGRA. However, considering the long duration of symptoms of the index patient, even if the baseline tests had been conducted, some may have already converted at the time.

A final limitation is that this study was based on the observation in a single nursing school. However, we believe the results can be extrapolated to other countries with a medium burden of TB similar to Japan.

In congregate settings, such as schools, administrators should be vigilant against TB to prevent outbreaks. Since it is difficult to distinguish cough caused by TB from cough due to asthma or other illnesses, physicians should take sputum samples for acid-fast bacilli tests from those who have persistent cough more than two weeks to minimize a diagnostic delay. In a country where BCG coverage is high, IGRA, rather than TST, should be used for screening in TB contact investigations. In congregate settings, the scope of contact investigation may have to be expanded to detect TB among those who had brief contact with the index patient.
